# Comment on ‘Effect of hypertension on clinical outcomes in ischemic colitis’

**DOI:** 10.1080/07853890.2025.2554922

**Published:** 2025-09-04

**Authors:** Tianchen Lin, Xiaobo Shen, Renhua Yang

**Affiliations:** a Affiliated Hangzhou First People’s Hospital, School of Medicine, Westlake University, Hangzhou, Zhejiang, China; b Huzhou Central Hospital, Affiliated Huzhou Hospital, Zhejiang University School of Medicine, Huzhou, Zhejiang, China

To the Editor

We read with great interest the article by Zhang et al. [1] examining the impact of hypertension on clinical outcomes in ischemic colitis (IC). The authors concluded that hypertension aggravated abdominal pain and endoscopic findings but did not significantly influence mortality, surgery, or hospital stay. While this work contributes important data, we wish to raise several methodological considerations.

First, optimization of clinical endpoint selection. Using mortality and surgical rate as the primary endpoints may lack sensitivity. The typical outcome of IC shows a bimodal distribution: 80% of patients can be relieved by conservative treatment (such as papaverine vasodilation), while 20% require surgical intervention [2]. Suggest adding the following indicators: (1) Mucosal repair time (evaluated through a series of colonoscopes). (2) Efficiency of gut microbiota reconstruction (monitored by fecal metagenomics). (3) Readmission rate (reflecting the progression of chronic ischemia).

Second, the definition of hypertension was based on medical history without stratification by disease duration or control status. Prior studies indicate that uncontrolled or long-standing hypertension may exert different pathophysiological effects than well-controlled disease. Future research should differentiate between controlled and uncontrolled hypertension to clarify their respective roles in IC.

Third, standardization challenges in endoscopic evaluation. The article mentions that the endoscopic manifestations of the hypertension group are more severe, but does not indicate whether the differences in the use of antiplatelet/anticoagulant drugs are controlled - these drugs are commonly used in cardiovascular disease patients and may exacerbate mucosal bleeding symptoms. It is recommended to adopt an improved IC endoscopic grading system (such as Marston grading) and record recent medication history to reduce evaluation bias [3].

What’s more, the proposed mechanism—that hypertension exacerbates ischemia *via* vascular dysfunction—is plausible but not directly tested. Integration of vascular and inflammatory biomarkers (e.g. endothelial adhesion molecules, C-reactive protein) or imaging evidence of mesenteric atherosclerosis would strengthen causal inference [4].

Finally, the study may have been underpowered to detect differences in hard outcomes. Event rates were low, with mortality of only 2.2% vs. 0.8% between groups. A larger, multicenter cohort or meta-analysis would be more suitable to evaluate associations with rare but clinically significant outcomes. Potential confounding by age and comorbidities deserves attention. The hypertensive group was significantly older and had a higher prevalence of coronary artery disease. Both factors are independently associated with IC prognosis [5]. Although multivariate analysis was performed, residual confounding may persist, particularly given the modest sample size.

In conclusion, Zhang et al. highlight a potentially important link between hypertension and symptom burden in IC. We believe that prospective, multicenter studies accounting for hypertension control and incorporating mechanistic biomarkers are needed to validate and expand upon these findings.

## Data Availability

Data sharing is not applicable to this article as no new data were created or analyzed in this study.
